# Evaluation of Neonatal Cardiotoxicity Following Maternal Green Tea Extract Consumption During Pregnancy: An Experimental Rat Study on the Cytochrome c/Caspase-9/Caspase-3 Pathway

**DOI:** 10.3390/medicina62050939

**Published:** 2026-05-12

**Authors:** Oya Sayın, Selda İldan Çalım, Ayşe Çigel, Seren Gülşen Gürgen

**Affiliations:** 1 Department of Medical Laboratory Techniques, Vocational School of Health Services, Dokuz Eylül University, 35330 Izmir, Turkey; 2 Department of Midwifery, Faculty of Health Sciences, Manisa Celal Bayar University, 45140 Manisa, Turkey; selda.ildan@cbu.edu.tr; 3 Department of Physiology, Faculty of Medicine, Izmir Democracy University, 35290 Izmir, Turkey; ayse.cigel@idu.edu.tr; 4 Department of Histology and Embryology, Vocational School of Health Services, Manisa Celal Bayar University, 45140 Manisa, Turkey; serengurgen@gmail.com

**Keywords:** green tea, prenatal exposure, neonatal heart, apoptosis, caspase-3, mitochondrial pathway

## Abstract

*Background and Objectives:* Green tea is known for its powerful antioxidant properties. However, the effects of green tea consumption during pregnancy on neonatal development and the mechanisms of these effects are not fully understood. The aim of this study was to investigate potential damage to atrial cardiomyocytes of newborn rat pups whose mothers received green tea during pregnancy and to elucidate the apoptotic mechanisms underlying this possible damage. *Materials and Methods:* Wistar albino rats (weighing 200–220 g, 10 weeks old) were used in this study. Following the confirmation of pregnancy, rats were randomly assigned to groups, and the experimental group was administered green tea by oral gavage at a dose of 50 mg/kg per day for 21 days. Atrial cardiomyocytes and mitral valve cells from newborn pups (postnatal day 1) were obtained and evaluated immunohistochemically for cytochrome c, caspase-9, and caspase-3 expression. *Results:* TUNEL analysis revealed a significant increase in DNA fragmentation in the green tea group, with the median number of apoptotic cells per region of interest (ROI) rising from 5.5 to 24.5 in atrial cardiomyocytes (*p* < 0.001), and from 2.0 to 10.0 in mitral valve cells (*p* < 0.05). Immunohistochemically, the control group showed faint-to-weak basal immunoreactivity of cytochrome c and caspase-3, and weak-to-moderate expression of caspase-9. In the green tea group, caspase-3 immunoreactivity was moderate, while cytochrome c and caspase-9 immunoreactivity were significantly higher. Quantitative HSCORE analysis confirmed significant elevations in atrial cardiomyocytes for cytochrome c (from 65.0 to 210.0; *p* < 0.001), caspase-9 (from 85.0 to 140.0; *p* < 0.001), and caspase-3 (from 60.0 to 120.5; *p* < 0.001). Similar statistically significant increases were observed across all corresponding markers in the mitral valve cells (*p* < 0.05). Overall, the induction of apoptosis was notably more pronounced in atrial cardiomyocytes than in mitral valve cells. *Conclusions:* Our findings suggest that the mechanism of potential damage in atrial cardiomyocytes of newborn rat pups is associated with mitochondria-mediated apoptosis, potentially triggered by activation of the cytochrome c, caspase-9 and caspase-3 axis. These results highlight the importance of exercising caution regarding the consumption of green tea supplements during pregnancy. Further studies are needed to correlate these preliminary neonatal observations with clinical outcomes.

## 1. Introduction

Polyphenols are the main active compounds found in tea. Green tea is obtained from the leaves of the Camellia sinensis plant and contains catechins such as epigallocatechin-3-gallate (EGCG), epigallocatechin, epicatechin-3-gallate and epicatechin, gallocatechins and gallocatechin gallate. EGCG is the primary catechin in tea, accounting for 50–70% of total catechins; consequently, most research has focused on this compound. Cell culture, human, and animal studies provide ample evidence showing that tea polyphenols have beneficial effects against various pathological conditions, including cancer, diabetes, and cardiovascular diseases [1,2,3,4,5,6,7,8,9]. However, recent toxicological studies report that the effects of EGCG are double-edged depending on exposure dose and physiological state. EGCG, especially at high doses, may acquire pro-oxidant properties rather than antioxidant effects [10]. Lambert et al. [10] reported that high-dose oral EGCG intake caused hepatotoxicity in mice by triggering mitochondrial dysfunction and oxidative stress. Cai et al. [11] suggested that high oral doses of EGCG may induce cardiac fibrosis. Furthermore, it has been shown that consuming four to nine cups of green tea per day increases the risk of lung cancer in humans [12] and causes teratogenicity such as anencephaly and neural tube defects [13]. Abdel Rasheed et al. [14] reported that administration of EGCG to diabetic mice exacerbated nephrotoxic damage by increasing inflammatory cytokines in kidney tissue. Furthermore, EGCG use during pregnancy has been shown to inhibit transcription of various genes by altering promoter methylation levels, thereby causing EGCG-induced cytotoxicity and tissue damage [15,16]. Studies have shown that green tea administered to pregnant rats causes immunohistochemical degenerative changes in the brain cortex and liver toxicity in the fetus [17,18]. These studies highlight not only the toxic effects observed in adults but also the effects of maternal ingestion of green tea on the developing fetus, particularly on critical organs undergoing organogenesis that can be exposed via substances crossing the placental barrier.

Apoptotic cell death occurs via two main pathways: the mitochondrial (intrinsic) pathway and the death-receptor (extrinsic) pathway. Mitochondrial damage causes release of cytochrome c into the cytoplasm. Cytochrome c binds to and activates Apaf-1. Upon addition of ATP, an apoptosome is formed. This complex leads to the activation of procaspase-9, which in turn becomes the active caspase-9. Caspase-9, in turn, initiates the activation of a number of downstream effector caspases, including caspase-3. Previous studies have stated that oxidative damage in cardiac cells triggers the release of cytochrome c into the cytosol, which activates the caspase-9 and caspase-3 enzymes, leading to cell death [19,20]. Furthermore, Ren et al. [21] emphasized that environmental toxins similarly increase ROS production, leading to cardiac developmental defects and disrupting mitochondrial integrity. Zhang et al. [15] demonstrated that prenatal EGCG exposure caused a decrease in cardiac fiber content and an increase in myocardial apoptosis in adult males.

The literature lacks sufficient information regarding the effects of maternal green tea consumption during pregnancy on neonatal heart tissue and the underlying molecular mechanisms. We hypothesized that intrauterine green tea exposure would activate the mitochondrial intrinsic apoptosis pathway in neonatal atrial cardiomyocytes and mitral valve cells, leading to irreversible cell loss. Accordingly, the aim of this study is to investigate the histopathological effects of exposure to green tea extract during pregnancy on neonatal rat atrial cardiomyocytes and mitral valve cells and to elucidate the molecular mechanisms underlying possible cardiotoxicity by assessing the mitochondrial apoptosis markers (cytochrome c, caspase-9, caspase-3) and DNA damage (TUNEL).

## 2. Materials and Methods

### 2.1. Experimental Animals

In this study, 18 female Wistar albino rats were used. All animals were housed at the Celal Bayar University Experimental Animal Application and Research Center (approval number: 2015/77.637.435-11, date of approval: 21 January 2015). To isolate the specific toxicological effects of green tea extract and account for the baseline physiological apoptosis occurring during neonatal development, all environmental variables were strictly synchronized for both control and experimental groups. These standardized conditions included an ambient temperature of 22 ± 2 °C, humidity of 55 ± 5%, and a 12 h light (07:00–19:00)/12 h dark (19:00–07:00) cycle. The animals were fed standard rat pellet feed and were provided with tap water *ad libitum*. Rats were checked once a day to detect changes in their health. All experimental procedures were conducted in strict compliance with the European Communities Council Directive of November 24, 1986 (86/609/EEC), and ethical approval was obtained from the Celal Bayar University Animal Care Committee.

### 2.2. Experimental Protocol

Female Wistar albino rats were mated with adult male rats. The presence of sperm in the vaginal smear or the detection of a vaginal plug was considered day 0 of gestation. Confirmed-pregnant rats (*n* = 18 in total) were divided into control and experimental groups using a simple randomization method (e.g., drawing lots): Group 1, the control group (standard diet and water, *n* = 9), and Group 2, the green tea group (standard diet and water supplemented with 50 mg/kg green tea extract, administered via oral gavage for 21 days, *n* = 9). After a 21-day gestation period, 2 newborn pups were randomly selected from each mother. Consequently, a total of 36 neonatal rat pups were evaluated: 18 pups in the control group and 18 pups in the green tea group. The pups were sacrificed on postnatal day 1 via intraperitoneal injection of ketamine (90 mg/kg) and xylazine (5 mg/kg). Heart tissues were removed from neonatal rats, fixed in 10% formaldehyde for 48 h, and embedded in paraffin blocks according to routine protocols. A microtome was used to obtain 5 μm sections, which were then subjected to immunohistochemical and TUNEL staining. Serial sections (5 μm thick) were obtained from each tissue block to allow for direct and comparative molecular evaluation of the same anatomical regions. This approach enabled the analysis of the expression of different apoptotic markers (cytochrome c, caspase-9, and caspase-3) in successive layers of the same cardiac units, including the atrial wall and mitral valve. Furthermore, evaluator blinding was rigorously applied during the data collection and outcome evaluation phases to minimize observer bias. The researchers performing the histological and immunohistochemical analyses were completely unaware of group assignments, as all samples were numerically coded before evaluation. To further ensure the objectivity and reliability of the semi-quantitative scoring, all slides were independently reviewed by two experienced histologists using a blinding method. High inter-observer reliability was found, and a high degree of correlation (r > 0.90) was observed. Minor discrepancies in the scoring were resolved through a joint reassessment under a multi-headed microscope to reach a final consensus. The cages used were numbered to minimize potential confounding factors such as the order of treatments and measurements or animal/cage location.

Criteria for including animals in the experimental protocol:

• A young adult female rat weighing 200–220 g, 10 weeks old.
• Female rats with confirmed pregnancies.

Criteria for excluding animals from the experimental protocol:

• The veterinarian deems the animal unsuitable for the study (humane endpoints)
• Weight loss exceeding 15% of body weight.
• Inadequate food and water intake.
• Significantly reduced response to stimuli.
• Reductions in the animal’s mobility.

### 2.3. Green Tea Extract Preparation

The green tea leaves used for extraction were obtained from a commercial supplier (Caykur, General Directorate of Tea Enterprises, Rize, Turkey). One hundred grams of dried green tea leaves were ground in a laboratory blender (model no. 64825; Merck, Darmstadt, Germany) and extracted with 1000 mL of distilled water at 35 °C for 1 h.

The extract was filtered and centrifuged at 3000 rpm for 15 min. The supernatant was collected; the remaining pellet was mixed with distilled water, extracted again at 35 °C, and centrifuged. The supernatant was collected, subjected to high vacuum, and stored at −20 °C until use. It is well documented that high temperatures cause thermal degradation and epimerization of heat-sensitive bioactive polyphenols (especially catechins such as EGCG) [22,23]. Therefore, to maximize the yield of antioxidant compounds, the extraction process was performed according to the protocol of Abdel-Majeed et al. [24]. Green tea extract was administered via oral gavage at a dose of 50 mg/kg of maternal body weight. The extract was dissolved in distilled water, and the administration volume was standardized to 5 mL/kg of body weight (1 mL solution for a 200 g rat) to ensure accurate and safe dosing.

### 2.4. TUNEL Method

Apoptotic cells were detected using the terminal deoxynucleotidyl transferase dUTP nick end labeling (TUNEL) assay (ApopTag Plus Peroxidase In Situ S7101, Chemicon International, Temecula, CA, USA). Tissue sections (5 μm) were deparaffinized at 60 °C for 1 h, then treated with xylene and a graded ethanol series (100%, 95%, 85%, 70%, 60%) to rehydrate. After washing with distilled water, sections were incubated with Proteinase K (20 μg/mL) for 10 min, then washed with PBS. Endogenous peroxidase activity was then inhibited with 3% hydrogen peroxide for 15 min. The sections were washed with PBS and then incubated in equilibration buffer for 10–15 min, followed by incubation in the TdT enzyme reaction mixture for 60 min, at 37 °C in a humidified chamber. The reaction was then stopped using a stop/wash buffer for 10 min. TUNEL-positive cells were detected by incubation with anti-digoxigenin conjugate for 45 min, followed by DAB chromogen staining and the subsequent application of Methyl Green as a background stain for 5 min. The stained slides were passed through an alcohol series and then left in xylene for 20 min to clear them. The preparations were then covered with Entellan and coverslips, and photographs were taken using a CX41 bright microscope (Olympus, Tokyo, Japan). For quantitative analysis, five randomly selected fields per section were evaluated (approximately 100 cells per field). All images were captured and analyzed at 400× magnification. At this magnification, the estimated Region of Interest (ROI) for each field was approximately 0.15 mm^2^. Cells showing distinct brown nuclear staining were identified as TUNEL-positive (apoptotic). The apoptotic index was calculated as the percentage of TUNEL-positive cells.

### 2.5. Immunohistochemical Methods

Tissue sections (5 μm thick) were deparaffinized in xylene and rehydrated through a graded ethanol series. Then, sections were treated with 3% hydrogen peroxide ( LabVision, Fremont, CA, USA) for 5 min to inhibit endogenous peroxidase activity in the tissue. After washing with phosphate-buffered saline (PBS), sections were incubated with a blocking solution (Invitrogen, Carlsbad, CA, USA) for 1 h at room temperature. Subsequently, sections were incubated overnight with primary rabbit polyclonal antibodies against cytochrome c (SC-13156, Santa Cruz Biotechnology, Dallas, TX, USA), caspase-9 (SC-56073, Santa Cruz Biotechnology, Dallas, TX, USA) and caspase-3 (SC-56053, Santa Cruz Biotechnology, Dallas, TX, USA, each diluted 1:100. The sections were then washed three times with PBS and incubated with a biotinylated secondary antibody and streptavidin-conjugated horseradish peroxidase (HRP) for 30 min (Invitrogen, Carlsbad, CA, USA) ). The reaction was visualized using DAB (3,3’-diaminobenzidine, Thermo Scientific, Fremont, CA, USA). Finally, sections were counterstained with Mayer’s hematoxylin and mounted. The obtained preparations were examined and photographed under a research microscope (Olympus CX31RTSF, Tokyo, Japan), and cell counts were made on digital images transferred to a computer using the ToupView image processing software (version 4.11, ToupTek Microsystem, No: V3.0-20180809, Hangzhou, China) application. During the immunohistochemical staining protocol, procedural controls were rigorously applied to verify antibody specificity and ensure the overall reliability of the test. For negative controls, primary antibodies were removed and replaced with phosphate-buffered saline (PBS) or non-immune serum. As expected, the complete absence of immunostaining was observed in these sections. Since these validation steps were rigorously performed as routine internal quality controls to verify batch specificity prior to the main evaluation, the control slides were evaluated directly by light microscopy by the investigators and were not photographically archived. However, the consistent absence of background staining in these internal controls confidently confirms the specificity of the primary antibodies and the accuracy of the baseline results observed in our study. The staining intensities of the slides prepared according to the immunohistochemical protocol were scored semi-quantitatively. Evaluations were performed on 5 randomly selected fields per tissue section at X400 magnification (ROI: 0.15 mm^2^). Staining intensity was scored semi-quantitatively as 0 (no staining), 1 (+, weak immunoreactivity), 2 (++ moderate immunoreactivity), and 3 (+++, strong immunoreactivity). The percentage of positive staining was scored by comparing immunoreactive cells or structures to the total number of cells or structures: 1 (0–10%, focal), 2 (11–50%, regional), and 3 (51–100%, diffuse). A composite score for each area was calculated using the formula Σ Pi × (i + 1), where Pi represents the percentage score and i is the staining intensity. This semi-quantitative assessment, validated by McCarty et al. [25], allows for a reliable evaluation of total protein expression by integrating both staining intensity and distribution. The results were aggregated to yield a single H-score value for each slide.

### 2.6. Statistical Analysis

Data were analyzed using SPSS for Windows version 15. The normality of data distribution was assessed using the Shapiro–Wilk test. As the results indicated that the data were not normally distributed (*p* < 0.05), non-parametric tests were applied. Comparisons between two independent groups were performed using the Mann–Whitney U test. Descriptive statistics were presented as median and interquartile range (Q1–Q3). All statistical tests were two-tailed. A *p*-value < 0.05 was considered statistically significant, and exact *p*-values are reported in the tables. Effect sizes (r) were calculated for the Mann–Whitney U test using the formula r = Z/√N [26]. The magnitude of the effect size was interpreted as small (0.10 ≤ r < 0.30), medium (0.30 ≤ r < 0.50), and large (r ≥ 0.50). Interobserver reliability between the two histologists was evaluated using Spearman’s rank correlation analysis.

## 3. Results

### 3.1. DNA Fragmentation and Apoptotic Index Findings (TUNEL)

**Figure 1:**
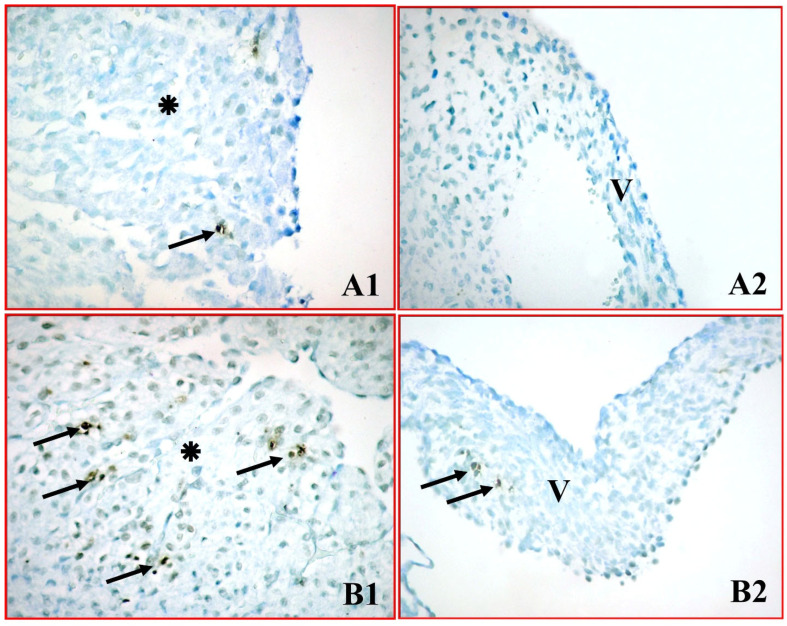
TUNEL immunoreactivity in neonatal atrial cardiomyocytes and mitral valve cells. Control group (**A1**,**A2**) shows normal integrity with few positive cells. Green tea group (**B1**,**B2**) shows widespread apoptotic cells, indicating significant DNA fragmentation. ✴: Atrial cardiomyocytes, V: Mitral valve cells, ➞: TUNEL positive cell. (×400).

The presence of apoptosis was evaluated in neonatal atrial cardiomyocytes and mitral valve cells using the TUNEL method, which indicates DNA fragmentation. In atrial cardiomyocytes from the control group, normal histological integrity was maintained, and only a few TUNEL-positive cells were present, consistent with the usual physiological cycle (Figure 1(A1,A2)).

**Figure 2:**
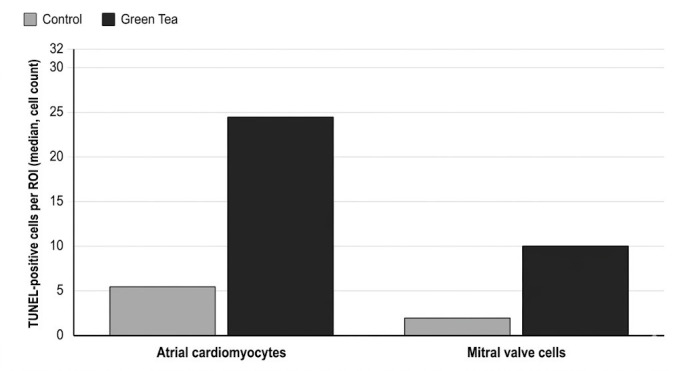
TUNEL-positive cell counts per region of interest (ROI) in neonatal cardiomyocytes and mitral valve cells. The green tea group shows a significant increase in apoptotic cells compared to the control group in both atrial cardiomyocytes (*p* < 0.001) and mitral valve cells (*p* < 0.05). Data are presented as Median (Q1–Q3).

In contrast, in the experimental group exposed to 50 mg/kg green tea extract during pregnancy, widespread TUNEL positivity was detected, characterized by intense brown staining, especially in atrial cardiomyocytes (Figure 1(B1,B2)). Quantitative analysis confirmed this observation; the median number of TUNEL-positive cells per ROI in atrial cardiomyocytes significantly increased from 5.5 (4.0–7.25) in the control group to 24.5 (22.0–27.0) in the green tea group (*p* < 0.001). A similar, highly significant increase was observed in the mitral valve cells, with the median value rising from 2.0 (1.0–3.0) to 10.0 (9.0–11.5) (*p* < 0.05) (Table 1, Figure 2).

### 3.2. Immunohistochemical Findings

**Figure 3:**
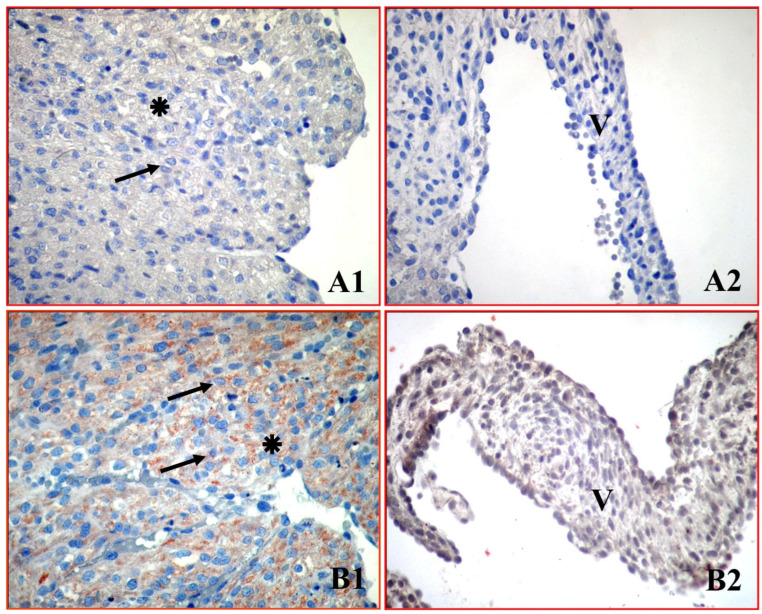
Cytochrome c immunoreactivity in neonatal atrial cardiomyocytes and mitral valve cells. Control group (**A1**,**A2**) shows faint-to-weak basal immunoreactivity. Green tea group (**B1**,**B2**) exhibits intense brown cytoplasmic staining, indicating significant cytochrome c release. ✴: Atrial cardiomyocytes, V: Mitral valve cells, ➔: Immunopositive cardiomyocytes. (×400).

In the control group, cytochrome c exhibited faint-to-weak basal immunoreactivity in atrial cardiomyocytes and mitral valve cells (Figure 3(A1,A2)). In contrast, the green tea group exhibited strong cytochrome c immunopositivity in the cytoplasm of atrial cardiomyocytes and moderate immunoreactivity in the mitral valve cells (Figure 3(B1,B2)).

Quantitative HSCORE analysis confirmed that cytochrome c expression was significantly increased in atrial cardiomyocytes and mitral valve cells in the green tea group compared to the control group (Table 1). Specifically, in atrial cardiomyocytes the median cytochrome c HSCORE increased from 65.0 (63.0–68.0) in the control group to 210.0 (208.0–213.0) in the green tea group (*p* < 0.001). Similarly, in the mitral valve cells, the median HSCORE increased from 55.0 (52.0–57.0) to 121.0 (119.0–124.0) (*p* < 0.05).

**Figure 4:**
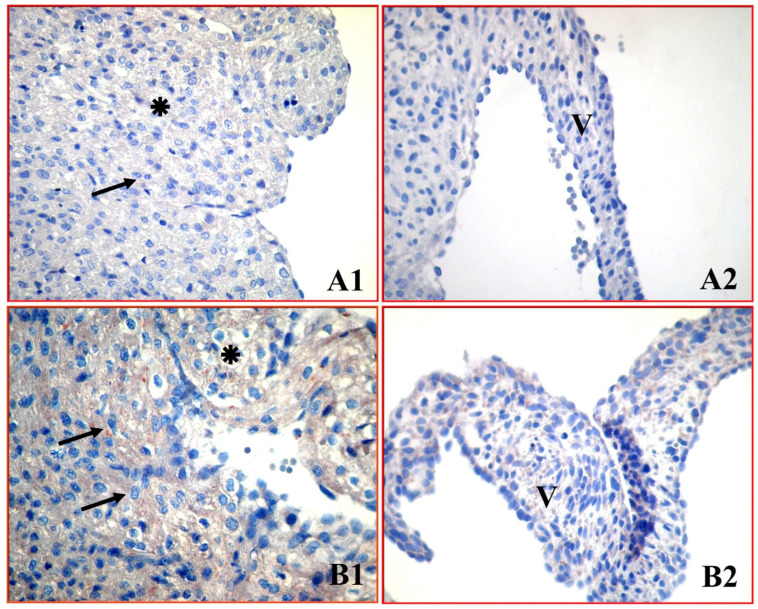
Caspase-9 immunoreactivity in neonatal atrial cardiomyocytes and mitral valve cells. Control group (**A1**,**A2**) shows weak-to-moderate basal expression. Green tea group (**B1**,**B2**) shows a significant increase in staining intensity, indicating activation of the mitochondrial apoptosis pathway. ✴: Atrial cardiomyocytes, V: Mitral valve cells, ➔: Immunopositive cardiomyocytes. (×400).

Immunoreactivity of caspase-9 ranged from weak to moderate in atrial cardiomyocytes and mitral valve cells in the control group (Figure 4(A1,A2)); however, in the green tea group, its expression showed a significant increase, paralleling the rise in cytochrome c (Figure 4(B1,B2)).

Quantitative HSCORE analysis revealed a significant increase in caspase-9 expression in the green tea group compared to the control group (Table 1). In atrial cardiomyocytes, median caspase-9 HSCORE values increased from 85.0 (82.0–87.0) to 140.0 (138.0–142.0) (*p* < 0.001). In the mitral valve cells, median values increased from 64.5 (62.0–67.0) to 80.0 (79.0–83.0) (*p* < 0.05) (Table 1).

**Figure 5:**
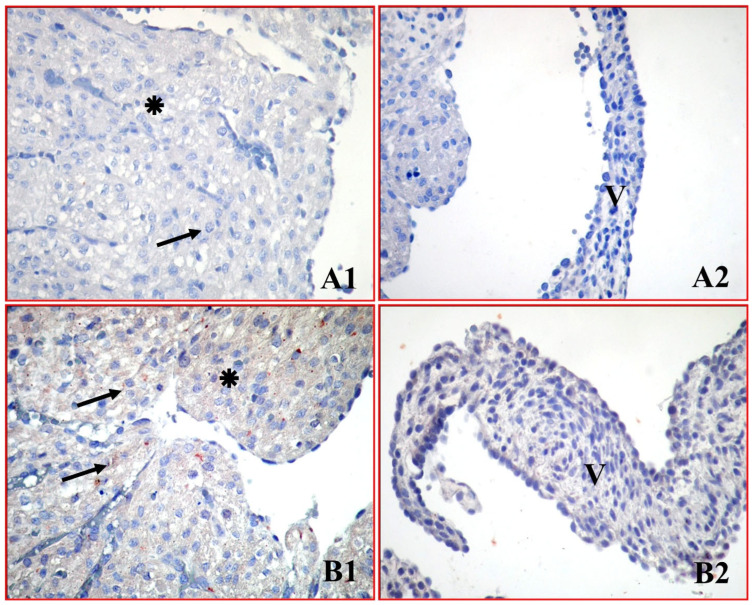
Caspase-3 immunoreactivity in neonatal atrial cardiomyocytes and mitral valve cells. Control group (**A1**,**A2**) shows faint-to-weak basal immunoreactivity. Green tea group (**B1**,**B2**) shows a moderate increase in staining, indicating the activation of the effector caspase. ✴: Atrial cardiomyocytes, V: Mitral valve cells, ➔: Immunopositive cardiomyocytes. (×400).

Immunohistochemical evaluation of caspase-3 revealed faint-to-weak basal immunoreactivity in the control group (Figure 5(A1,A2)), while the number of immunopositive cells and staining intensity were moderately increased especially in atrial cardiomyocytes in the green tea group (Figure 5(B1,B2)).

Quantitative HSCORE analysis demonstrated a significant increase in the green tea group compared to the control group (Table 1). Specifically, in the atrial cardiomyocytes, the median caspase-3 HSCORE increased from 60.0 (58.0–62.0) in the control group to 120.5 (118.0–123.0) in the green tea group (*p* < 0.001). Similarly, in the mitral valve cells, the median HSCORE significantly increased from 53.0 (51.0–55.0) to 84.5 (82.0–87.0) (*p* < 0.05).

**Figure 6:**
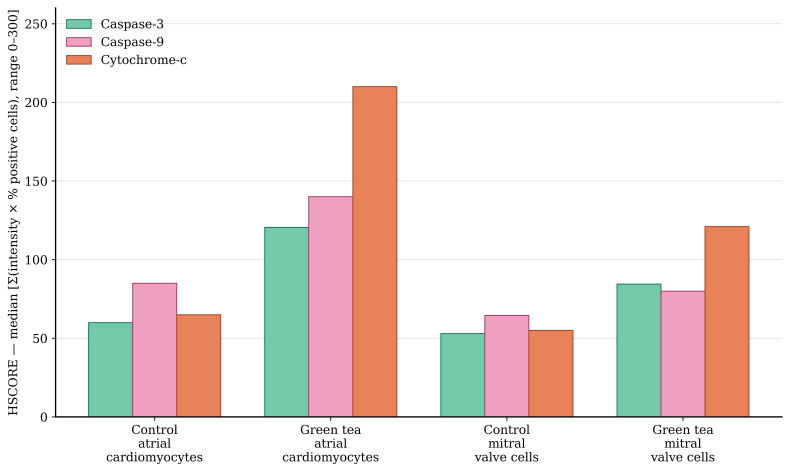
Immunohistochemical HSCORE for caspase-3, caspase-9, and cytochrome c in neonatal cardiomyocytes and mitral valve cells. The green tea group exhibits significantly increased HSCOREs for all markers compared to the control group in both atrial cardiomyocytes (*p* < 0.001) and mitral valve cells (*p* < 0.05).

The comparative HSCORE values for all immunohistochemical markers (cytochrome c, caspase-9, and caspase-3), demonstrating the significant elevation of apoptotic proteins in the green tea group, are graphically summarized in Figure 6.

The effect sizes for all non-parametric comparisons (Mann–Whitney U test) were large (r = 0.855–0.859, calculated using the formula r = Z/√N). This indicates that the effect size of the apoptotic differences induced by green tea extract is quite large.

## 4. Discussion

This study demonstrated that a dose of 50 mg/kg/day of green tea extract, administered maternally during pregnancy, triggered intrinsic (mitochondrial) apoptosis in the heart tissue of newborn rats, leading to cytochrome c release and subsequent activation of caspase-9 and caspase-3.

It is well established that a certain degree of apoptosis occurs physiologically in the neonatal heart as part of normal postnatal remodeling. However, in the present study, the markedly increased levels of TUNEL-positive cells and pro-apoptotic markers in the green tea group, compared to the control group under identical experimental conditions, strongly suggest that this increase exceeds physiological apoptosis and reflects a treatment-related effect.

The main component of green tea, epigallocatechin-3-gallate, has been reported to have antidiabetic, anticancer, antiatherosclerotic, antioxidant, anti-inflammatory and protective properties against cardiovascular diseases [1,27,28]. Maternal green tea consumption has been shown to protect against dyslipidemia, glucose intolerance, and fat accumulation in adult offspring fed a high-fat diet after weaning [29]. Green tea intake during lactation reduces tubulointerstitial fibrosis and macrophage infiltration in the kidneys of adult offspring programmed by maternal protein restriction and fed a high-fat diet by downregulating epigenetic modulators [30]. Green tea and its main component, EGCG, which have many health benefits, have experienced increased consumption in recent years. The benefits of tea stem from the flavonoids it contains. However, these flavonoids can have some harmful effects on human health when consumed in excess of certain limits.

Morita et al. [31] administered green tea catechin orally to female rats at doses of 0, 200, 600, and 2000 mg/kg/day between days 6 and 17 of gestation and observed no green tea-related deaths or macroscopic findings. In studies showing no observable adverse effects, a dose level of 200 mg/kg/day of green tea was considered toxic for the mother; for the fetus, a dose of 2000 mg/kg/day was accepted as toxic. It has been reported that pregnant rats given green tea at doses of 40, 200, and 500 mg/kg per day between days 6 and 17 did not adversely affect fetal development [32]. On the other hand, Correa et al. [13] reported that the mother’s consumption of 3 cups of green tea per day during the periconceptional period caused developmental neural tube defects in the fetus. The literature has shown that the effect of EGCG can vary depending on the dose and exposure period; indeed, at high doses or during sensitive periods, such as the prenatal period, EGCG can paradoxically cause tissue damage by inducing pro-oxidant and teratogenic effects [10,15]. Green tea polyphenols are widely recognized for their ROS-scavenging properties and antioxidant capacity in adult tissues and in various disease models [33]. However, this paradigm is reversed when it comes to fetal physiology. Compared to adult tissues, fetal tissues are not yet mature in terms of endogenous antioxidant enzyme systems, such as superoxide dismutase (SOD) and catalase, and are therefore extremely vulnerable to oxidative stress. In this context, the “Pro-oxidant Shift” theory, described by Stoeva et al. [34] and Lambert et al. [10], provides a fundamental mechanistic explanation for our findings. According to this theory, the well-established antioxidant properties of green tea polyphenols can paradoxically convert into cytotoxic pro-oxidant activity depending on variables such as high dosage, the presence of transition metal ions, and intracellular pH. High doses of EGCG, in particular, interact with transition metals (copper/iron) in intracellular redox cycles, undergoing auto-oxidation and paradoxically triggering the production of hydrogen peroxide (H_2_O_2_) and superoxide anion (O_2_^•−^). Our study shows that the hearts of the offspring are vulnerable to increased endogenous ROS production, with the antioxidant balance shifted towards degradation.

The mechanisms underlying the high-dose toxicity of EGCG have been attributed to various signaling pathways through studies conducted in different tissues. For example, Lambert et al. [10] demonstrated that high-dose oral EGCG administration in mice caused hepatotoxicity by triggering mitochondrial dysfunction and oxidative stress. Similarly, Cai et al. [11] reported that high-dose EGCG (500 and 1000 mg/kg/day) exerted cardiotoxic effects in mice by increasing the expression of fibrosis-related proteins, such as connective tissue growth factor and fibronectin. Khan et al. [1] reported that high doses of polyphenols can increase hydroxyl radical (•OH) production via the Fenton reaction by reducing intracellular copper ions. Fetal heart tissue is susceptible to this oxidative stress due to its abundant mitochondrial content and limited regenerative capacity.

Elborn et al. [17] evaluated the effects of exposure to different doses of green tea extract (200, 600 and 1000 mg/kg/day) during the organogenesis phase (days 6–15 of gestation) on central nervous system development in 20-day-old rat fetuses. They showed that green tea increased glial fibrillary acidic protein (GFAP) and decreased proliferating cell nuclear antigen (PCNA) in a dose-dependent manner, ultimately leading to degenerative changes in the cerebral cortex, cerebellum, and spinal cord. Zielinsky et al. [35] demonstrated clinically and experimentally that maternally consumed green tea inhibited prostaglandin synthesis in late-term lamb fetuses, leading to narrowing of the fetal ductus arteriosus.

Xia et al. [16] showed that EGCG exposure during pregnancy (1 mg/kg) led to histomorphological abnormalities in the uterine tissue of female mouse pups by activating the NF-κB (p65) signaling pathway. Zhang et al. [15] found that prenatal EGCG exposure significantly reduced heart mass in adult male mice. In their experimental model, they exposed the C57BL/6 mice to EGCG (3 μg/mL) intrauterinely for 16 days. They observed a significant decrease in the heart/body weight ratio in adult males, but no such decrease in adult females. They have attempted to explain this pathology through changes in the TGF-β1/SMAD signaling pathway. In adult male hearts, inhibition of the PI3K/AKT signaling pathway increases the expression of proapoptotic proteins such as BAX, cleaved caspase-3, and cleaved caspase-9, while decreasing the expression of antiapoptotic proteins such as BCL-2. In our study, we detected intense cytochrome c release and caspase-9 activation in cardiomyocytes of neonatal rat hearts exposed to 50 mg/kg green tea extract throughout gestation and demonstrated that the primary process initiating cardiotoxicity is intrinsic apoptosis, which begins with disruption of mitochondrial outer membrane integrity. Abdel Rasheed et al. [14] reported that EGCG exacerbated tubular damage in the kidney tissue of diabetic mice by increasing pro-inflammatory cytokines such as TNF-α and IL-1β. Zhao et al. [20] showed that oxidative stress in cardiomyocytes disrupts the mitochondrial membrane and that release of cytochrome c increases caspase-9 and caspase-3 activities, leading to cell death. A similar mechanistic relationship was demonstrated by Ozkaya et al. [19] in an amiodarone toxicity model. The researchers determined that oxidative damage caused by chronic drug exposure directly triggers the apoptosis mechanism; they proved with histopathological and immunohistochemical data that the increase in the activation of caspase-3 and the intense DNA fragmentation detected by the TUNEL method are the main causes of germ cell loss and tissue atrophy. In our previous study, we evaluated the effects of green tea consumption during pregnancy on the apoptosis marker CK-18 in the liver tissue of maternal and newborn rats and showed that green tea had no negative effect on the mother but induced CK-18 expression in the newborn [18]. These studies reveal that EGCG toxicity is mediated by oxidative stress, inflammation, and mitochondrial damage, but the mechanisms may vary depending on the tissue.

To our knowledge, no previous study has investigated the effect of green tea consumption during pregnancy on the molecular-level expression of apoptotic markers cytochrome c, caspase-9, and caspase-3 in cardiomyocytes and mitral valve cells. Our study elucidates the cardiotoxic mechanism of green tea during the prenatal period. In our study, a strong correlation between the marked increase in caspase-3 activation and TUNEL positivity suggests that neonatal cardiomyocytes are genetically predisposed to apoptosis. The intense TUNEL-positive cell death observed in the offspring of pregnant rats administered green tea is attributable to DNA damage in fetal cardiac tissue. In this process, the significant increase in cytochrome c and caspase-9 observed in the green tea group demonstrates that toxicity proceeds via an intrinsic pathway directly triggered by disruption of mitochondrial integrity, as reported by Lambert et al. [10]. In the final stage, a significant increase in caspase-3 levels indicates that mitochondrial damage completes the caspase cascade, leading to irreversible cardiomyocyte death.

Cardiomyocyte death is fundamentally caused by the loss of mitochondrial integrity. Under normal physiological conditions, the mitochondrial outer membrane prevents the release of pro-apoptotic factors into the cytosol. However, the high cytochrome c immunopositivity detected in our experimental group indicates irreversible permeabilization of the mitochondrial outer membrane. Abdel Rasheed et al. [14] reported that EGCG causes loss of tubular cells in a model of diabetic nephrotoxicity by increasing caspase-3 activation.

In our study, apoptosis was more pronounced in atrial cardiomyocytes than in mitral valve cells. This can be explained by the cells’ unique bioenergetic structure. Although atrial cardiomyocytes are known to exhibit endocrine properties, including the secretion of atrial natriuretic peptide (ANP), this functional characteristic is not expected to significantly influence the mitochondrial apoptosis pathways evaluated in the present study. Cardiomyocytes have the highest mitochondrial density in the body, enabling them to maintain their lifelong, uninterrupted contractile function; these organelles occupy approximately 30–40% of the cell volume [36,37,38]. In contrast, interstitial cells and fibroblasts found in the cardiac valve region have a much lower mitochondrial content [39]. Therefore, because the observed damage progresses directly via the ‘mitochondrial pathway’ and subsequent cytochrome c release, cardiomyocytes become the primary target. These muscle cells, which possess a rich mitochondrial pool, have a much larger ‘damage surface’ exposed to the oxidative stress induced by high doses of polyphenols, resulting in pronounced cytochrome c release into the cytosol and stronger activation of the caspase-9 and caspase-3 cascades than in other tissues. Consequently, in our study, lower levels of apoptosis were observed in mitral valve cells than in atrial cardiomyocytes.

Regarding the clinical context of prenatal exposure, women may unknowingly continue pre-pregnancy weight-loss regimens and high-dose green tea supplements during the first trimester, when organogenesis is most critical. Furthermore, the misconception that herbal products are inherently safe can lead many pregnant women to consume such extracts as antioxidant supplements or diuretics without medical supervision.

In this study, a dose of 50 mg/kg/day was selected. Using the body surface area normalization method, this dosage corresponds to a human equivalent dose (HED) of approximately 486 mg/day for an individual weighing 60 kg [40]. This amount is equivalent to consuming three 8 oz. cups of green tea per day [41]; however, it is crucial to distinguish between dietary intake and supplement exposure. This high concentration primarily reflects the daily doses provided by concentrated herbal dietary supplements or extracts, which are frequently used for weight control [42]. Therefore, the dose in our experimental model specifically represents a realistic exposure risk associated with concentrated supplement exposure, rather than ordinary dietary beverage consumption.

Reports in the literature indicate that green tea catechins cross the placental barrier from maternal plasma to the fetus and concentrate in specific organs such as the eyes and brain [43]. Green tea polyphenols exhibit cytoprotective and antioxidant effects in adults, yet they cause selective toxicity in fetal tissues. The fundamental reason for this paradoxical situation is that the fetal antioxidant defense system, particularly the activities of superoxide dismutase and catalase, is not yet sufficiently mature compared with that of adults [34]. Therefore, hydrogen peroxide (H_2_O_2_) and free radicals released as a result of the auto-oxidation of high doses of polyphenols cannot be adequately detoxified in fetal myocardial tissue [10]. This redox imbalance leads to mitochondrial damage and apoptosis observed in our study. The 50 mg/kg dose used in our study represents not only the amount of green tea consumed in the diet but also a high exposure risk posed by increasingly common concentrated herbal extracts and weight-loss supplements.

This study has several limitations that need to be considered. First, in terms of chemical and analytical scope, whole green tea extract was used instead of pure EGCG to simulate a real-world maternal exposure scenario. However, it is important to acknowledge that the total catechin content and EGCG load in green tea can vary significantly (ranging roughly from 9% to 18% of dry weight) depending on factors such as cultivar, shading, harvest season, and processing method [44,45]. While this approach provides a comprehensive overview of combined catechin toxicity, the lack of detailed chromatographic analysis (e.g., HPLC) to determine the precise composition of the extract used constitutes a limitation [46]. Consequently, the exact ratios of EGCG, caffeine, and other bioactive polyphenols in the administered extract remain unquantified. Future studies with standardized EGCG fractions are needed to identify the specific molecules responsible for the observed neonatal cardiotoxicity. Furthermore, although a standardized commercial tea was used, specific heavy metal and mycotoxin screenings and formal pharmacokinetic analyses (e.g., measurement of plasma and tissue catechin concentrations via LC-MS/MS) were not performed. To correlate our immunohistochemical findings with circulating specific catechin levels, these analytical methods will need to be incorporated into future research.

Secondly, there are some inherent limitations in terms of experimental model and statistical design. Interspecies differences in placental anatomy must be carefully considered when extrapolating these findings to humans. As emphasized by Furukawa et al. [47], the rat placenta features a three-layered hemotrichorial structure, which serves as a more complex biological barrier compared to the single-layered hemomonochorial human placenta. This critical anatomical difference implies that the transplacental transfer of biologically active polyphenols, such as EGCG, may be much more direct and efficient in humans. Consequently, the thinner human placental barrier could facilitate higher fetal exposure levels, potentially amplifying the neonatal cardiotoxic risks observed in our experimental model even at lower levels of maternal consumption. Furthermore, inherent morphological and functional differences between rodent and human neonatal hearts must be acknowledged, as these may influence the physiological response to polyphenolic stress [48]. Since the amount of green tea crossing the placental barrier was not measured in our study, future research including these analytical measurements will be needed to provide guidance from a translational medicine perspective. Finally, to minimize the known ‘litter effect’, we limited our sample size to two offspring per mother. However, analyzing these offspring as independent observations without nested statistical models remains a methodological limitation. Nevertheless, the highly significant apoptotic outcomes (*p* < 0.001) strongly suggest that the cardiotoxic effect of the green tea extract overshadowed any potential intra-litter variations. Further validation of these findings and better application to human pregnancies require future experimental designs incorporating mixed-effects models.

## 5. Conclusions

We believe that our study contributes to the literature by providing mechanistic insights into the effects of green tea extract on fetal heart development in a rat model. Our findings suggest that the observed changes in cardiac tissue are associated with mitochondria-mediated apoptosis, potentially involving the activation of the cytochrome c, caspase-9, and caspase-3 axis. In this context, our study indicates that, despite its well-known protective effects in adults, green tea extract may pose a potential cardiotoxic risk to the developing fetus and could interfere with mitochondrial integrity. Importantly, these findings must be interpreted within the context of concentrated supplement exposure—as reflected by our calculated Human Equivalent Dose—rather than standard dietary beverage consumption. While traditional green tea consumption as a brewed beverage may be safe, these data highlight that pregnant women should be strictly cautioned against the use of high-dose herbal extracts. This provides a preliminary molecular basis for future toxicological risk assessments in translational medicine.

## Figures and Tables

**Table 1:** Quantitative analysis of TUNEL-positive (Apoptotic Index) and Immunohistochemical (H-SCORE) values in neonatal atrial cardiomyocytes and mitral valve cells. Data are presented as Median (Q1–Q3). HSCORE = Σ(staining intensity × percentage of positive cells), range 0–300. Q1–Q3 values based on Tukey’s hinges. Mann–Whitney U test; exact significance (2-tailed). n = 18 per group.

Parameter	Tissue	Control Median (Q1–Q3)	Green Tea Median (Q1–Q3)	U	Z	*p*
TUNEL (cells/ROI)	Atrial cardiomyocytes	5.5 (4.0–7.25)	24.5 (22.0–27.0)	0.000	−5.138	<0.001
	Mitral valve cells	2.0 (1.0–3.0)	10.0 (9.0–11.5)	0.000	−5.154	<0.05
Cytochrome c (HSCORE)	Atrial cardiomyocytes	65.0 (63.0–68.0)	210.0 (208.0–213.0)	0.000	−5.130	<0.001
	Mitral valve cells	55.0 (52.0–57.0)	121.0 (119.0–124.0)	0.000	−5.129	<0.05
Caspase-9 (HSCORE)	Atrial cardiomyocytes	85.0 (82.0–87.0)	140.0 (138.0–142.0)	0.000	−5.130	<0.001
	Mitral valve cells	64.5 (62.0–67.0)	80.0 (79.0–83.0)	0.000	−5.132	<0.05
Caspase-3 (HSCORE)	Atrial cardiomyocytes	60.0 (58.0–62.0)	120.5 (118.0–123.0)	0.000	−5.133	<0.001
	Mitral valve cells	53.0 (51.0–55.0)	84.5 (82.0–87.0)	0.000	−5.130	<0.05

## Data Availability

Data will be made available on request.
